# Energy Drinks and Atrial Fibrillation: An Unusual Case of Caution

**DOI:** 10.7759/cureus.10807

**Published:** 2020-10-05

**Authors:** Muhammad Hanif, Sana Saleem, Sidra Naz, FNU Sundas

**Affiliations:** 1 Internal Medicine, Khyber Medical College, Peshawar, PAK; 2 Cardiology, District Headquarters Teaching Hospital, Sargodha, PAK; 3 Internal Medicine, University of Health Sciences, Lahore, PAK

**Keywords:** cardiac arrhythmia, atrial fibrillation, energy drink

## Abstract

The most well-known type of cardiovascular arrhythmia in the United States and worldwide is atrial fibrillation (AF). Generally, 2.3 million individuals are determined to have AF in the United States. Energy drinks contain caffeine, taurine, theophylline, and sugars that enhance alertness. Consumption of energy drinks is mounting in the young population and has been associated with AF, regardless of the lack of conclusive evidence in the literature. This is a case report of a 22-year-old male without any significant cardiac history who was diagnosed with AF following intake of energy drinks.

## Introduction

The prevalence of atrial fibrillation (AF) increases with age, and young adults are at low risk (less than 1% of patients with AF are <60 years of age) [1]. Consumption of stimulants such as caffeine in young adults has been soaring and is a conceivable trigger of AF, as it increases the heart rate. Di Rocco et al. reported AF in two Caucasian adolescent males after excessive caffeine ingestion [2]. Similarly, three cases of AF with fast ventricular response after consuming energy drink were reported by Mattioli et al. All the patients were successfully cardioverted spontaneously and after pharmacological treatment [3]. Here, we present a case of 22-year-old male with no previous comorbidities who was diagnosed with AF after consuming energy drinks.

## Case presentation

**Figure 1:**
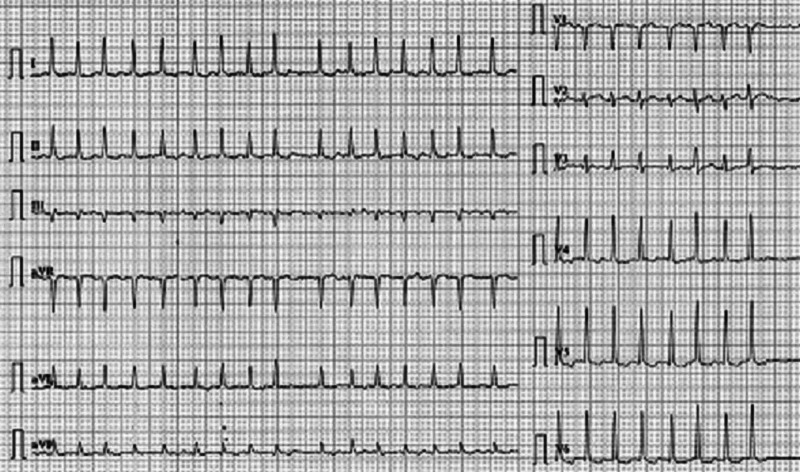
EKG showing atrial fibrillation with rapid ventricular response (ventricular rate: 150 beats per minute). EKG, electrocardiogram

A 22-year-old male student with no significant past medical or family history presented to the emergency department (ED) early in the morning with complaints of shortness of breath. He had consumed two energy drinks prior to his semester examination that day. Initially, on consuming the drink, the patient felt energetic and alert. However, after an hour, he started feeling short of breath and restless and vomited. The caffeine and taurine content of the drink was 111 mg. On examination, the patient appeared restless, with a heart rate in the 150s, a heartbeat that was irregular without murmurs, rubs, or gallops, and a blood pressure of 115/80 mmHg. An electrocardiogram (EKG) was obtained that showed AF with rapid ventricular response (Figure 1). Work-ups for thyroid functions and serum electrolytes (potassium, sodium, calcium, phosphate, and magnesium) were performed, the results of which were normal. Echocardiogram showed a structurally normal heart with an ejection fraction of 55%. Urine toxicology was sent, which revealed no toxic substance. The patient was admitted for further evaluation of his AF. Spontaneous resolution of the AF occurred during the observation. The patient was advised follow up after one month. An EKG and echocardiogram were repeated on the follow-up, which were normal.

## Discussion

Energy drinks are advertised across the world and are popular among young adults. According to self-repot surveys conducted by Seifert et al. [4], 30%-50% of adolescents and young adults consume energy drinks. The caffeine content varies from 50 mg to 500 mg per bottle [5]. Herbs, ginseng, and riboflavin are found in energy drinks such as Red Bull and Monster [6]. However, complications including insomnia, jitters, nervousness, high blood pressure, elevated heart rate, coronary artery spasm, coronary artery dissection, prolonged QT interval, and supraventricular arrhythmia can occur [7]. Few case reports provide evidence of AF, though a case of supraventricular tachycardia was reported in a 23-year-old woman without any cardiac medical history; the mechanism of this phenomenon is yet to be understood [8]. Caffeine is thought to play a role in neurohormonal stimulation and activation of the sympathetic autonomic nervous system [9]. Several recent studies have investigated the correlation between chronic caffeine consumption and AF; however, no significant increase in risk was established [10]. The amounts of caffeine, sugar, and sodium in energy drinks are much higher than those in sodas and other carbonated drinks [11]. Another possibility could be that the herbs in energy drinks are triggering arrhythmia. Further studies are required to determine any risk of energy drink consumption in prompting AF.

In this case report, this young adult consumed two bottles of energy drink and subsequently developed AF that spontaneously resolved to normal sinus rhythm, as seen on the EKG.

## Conclusions

Few cases of AF triggered by energy drink consumption have been reported, and the mechanism linking energy drink consumption and AF has yet to be determined. With the growing consumption of energy drinks, more clinical data are required. Physicians should be aware of the possible complications of cardiac arrhythmia associated with energy drink consumption, and people should be educated about such complications in the future.
